# Microtubule Dynamics Regulate Cyclic Stretch-Induced Cell Alignment in Human Airway Smooth Muscle Cells

**DOI:** 10.1371/journal.pone.0026384

**Published:** 2011-10-17

**Authors:** Masataka Morioka, Harikrishnan Parameswaran, Keiji Naruse, Masashi Kondo, Masahiro Sokabe, Yoshinori Hasegawa, Béla Suki, Satoru Ito

**Affiliations:** 1 Department of Respiratory Medicine, Nagoya University Graduate School of Medicine, Nagoya, Japan; 2 Department of Physiology, Nagoya University Graduate School of Medicine, Nagoya, Japan; 3 Department of Cardiovascular Physiology, Okayama University Graduate School of Medicine, Okayama, Japan; 4 Department of Biomedical Engineering, Boston University, Boston, Massachusetts, United States of America; Massachusetts Institute of Technology, United States of America

## Abstract

Microtubules are structural components of the cytoskeleton that determine cell shape, polarity, and motility in cooperation with the actin filaments. In order to determine the role of microtubules in cell alignment, human airway smooth muscle cells were exposed to cyclic uniaxial stretch. Human airway smooth muscle cells, cultured on type I collagen-coated elastic silicone membranes, were stretched uniaxially (20% in strain, 30 cycles/min) for 2 h. The population of airway smooth muscle cells which were originally oriented randomly aligned near perpendicular to the stretch axis in a time-dependent manner. However, when the cells treated with microtubule disruptors, nocodazole and colchicine, were subjected to the same cyclic uniaxial stretch, the cells failed to align. Lack of alignment was also observed for airway smooth muscle cells treated with a microtubule stabilizer, paclitaxel. To understand the intracellular mechanisms involved, we developed a computational model in which microtubule polymerization and attachment to focal adhesions were regulated by the preexisting tensile stress, pre-stress, on actin stress fibers. We demonstrate that microtubules play a central role in cell re-orientation when cells experience cyclic uniaxial stretching. Our findings further suggest that cell alignment and cytoskeletal reorganization in response to cyclic stretch results from the ability of the microtubule-stress fiber assembly to maintain a homeostatic strain on the stress fiber at focal adhesions. The mechanism of stretch-induced alignment we uncovered is likely involved in various airway functions as well as in the pathophysiology of airway remodeling in asthma.

## Introduction

Mechanical stretch has been found to affect a variety of cellular properties such as cell shape, motility, stiffness, contraction, orientation and cell alignment [1], [2], [3], [4], [5], [6], [7]. Airway smooth muscle (ASM) cells within airway walls are continuously exposed to anisotropic, cyclically varying mechanical forces through tidal stretching of the underlying extracellular matrix (ECM). In vivo, ASM cells wrap airways in helical fashion at an angle of about 75° with respect to the long axis of the airway [8], [9]. Because of this unique helical arrangement, the angle of orientation is a major factor that determines the extent to which airways constrict in response to ASM activation [10]. Therefore, the intracellular mechanisms by which cyclic stretch affects cell orientation and alignment are important in the normal functioning of the respiratory system as well as the pathogenesis of airway remodeling and hyper-responsiveness in asthma [11], [12].

When a population of randomly oriented cells is exposed to cyclic uniaxial stretch, the cells respond by aligning with their long axis in the direction of minimum strain [13], [14], [15]. Previous studies have attributed this phenomenon to the activation of Rho pathway which induces cytoskeletal remodeling specifically the formation of actin stress fibers in the direction of minimum strain and the turnover of focal adhesions [7], [14]. In an unstretched cell, the forces at a focal adhesion are borne not only by the actin stress fibers but also the microtubules – stiff, hollow, tubular structures that can rapidly polymerize and depolymerize at their free ends [16], [17], [18], [19], [20]. It was shown that disruption of microtubule polymerization blocks cell orientation induced by fluid shear stress in bovine aortic endothelial cells [21]. Nevertheless, the role of microtubules in determining the cell reorientation in response to cyclic stretch is not well understood. Since the alignment process involves changes in force balance and remodeling of focal adhesions [6], we hypothesized that microtubules contribute to the intracellular processes that drive stretch-induced orientation in ASM cells.

To test this hypothesis, we determined the alignment response together with the intracellular cytoskeletal structure induced by uniaxial stretch of human ASM (HASM) cells in culture before and after disruption or stabilization of microtubules. Additionally, to better understand the intracellular dynamics of individual cells that lead to cell alignment, we developed a computational model in which microtubule polymerization and attachment to focal adhesions is regulated by the preexisting tensile stress, pre-stress, on actin stress fibers. We demonstrate that microtubules contribute to the alignment of HASM cells subjected to cyclic uniaxial stretch. Our findings suggest that microtubules and stress fibers act in tandem to dynamically balance the applied stretch pattern by trying to reestablish a stable mechanical equilibrium.

## Materials and Methods

### Cell Culture

Primary cultures of normal human bronchial smooth muscle cells from multiple donors were obtained from Cambrex Co. (Walkersville, MD, USA). The cells were maintained in culture medium containing 5% fetal bovine serum (FBS), human recombinant epidermal growth factor (1 ng/ml), insulin (10 mg/ml), human recombinant fibroblast growth factor (2 ng/ml), gentamycin (50 mg/ml) and amphotericin B (0.05 mg/ml) (SmGM-2 BulletKit; Cambrex Co.) in an atmosphere of 5% CO_2_ and 95% air at 37°C. The cells retain expression of smooth muscle marker proteins such as α-smooth muscle actin, smooth muscle myosin heavy chain, and calponin [22].

### Application of Uniaxial Cyclic Stretch

The cells at the 4–7th passage were removed from the dish with 0.01% EDTA-0.02% trypsin and transferred to a 4 cm^2^ silicone chamber (2 cm long, 2 cm wide, and 1 cm deep) (STB-CH-04; Strex, Osaka, Japan) coated with type I collagen (Nitta-gelatin, Osaka, Japan) at a density of 2.0×10^4^ cells/cm^2^. A uniaxial sinusoidal stretch of 20% in strain at 30 cycle/min was applied using stretching apparatus driven by a computer-controlled stepping motor (ST-140; Strex) in an atmosphere of 5% CO_2_ and 95% air at 37°C as described previously [23], [24], [25]. Briefly, one end of the chamber was attached to a fixed frame, while the other end was attached to a movable frame. Other two sides were free to move. The movable frame was connected to a motor driven shaft whose amplitude and frequency of stretch was controlled by a programmable microcomputer. Twelve hours prior to stretching, cells were brought to a quiescent state by incubation in Dulbecco's modified Eagle's medium (DMEM)/F-12 culture medium (Invitrogen, Carlsbad, CA, USA) with 0.03% FBS. The silicon chamber had a 200-µM thick transparent bottom and the side walls were 4 mm thick to reduce compression of the chamber perpendicular to the direction of the stretch. The relative elongation of the silicone membrane was uniform across the whole membrane area, and lateral contraction did not exceed 3% at 20% stretch. The cells incubated under a static condition on the silicone chamber were used as a time-matched control. To determine whether microtubules play a role in the cyclic stretch-induced cell reorientation, effects of inhibitors of microtubule polymerization, nocodazole (Calbiochem, La Jolla, CA, USA) and colchicine (Calbiochem), or a microtubule stabilizer paclitaxel (Calbiochem) were examined. Either drug was applied to the cell culture medium 30-min prior to the application of cyclic stretch.

### Measurement of Cell Orientation

**Figure 1:**
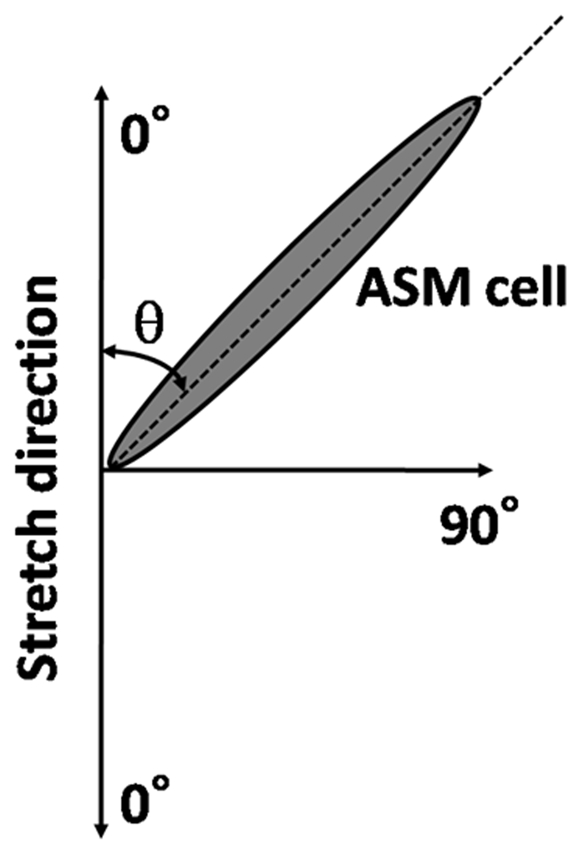
Schematic of how the angle of orientation (θ) of the long axis was measured. The angle is always between 0° and 90° with respect to the stretch axis (arrows).

Phase-contrast images of the cells were photographed using a ×20 objective (PM20; Olympus, Tokyo, Japan) with at least three arbitrarily selected visual fields of the microscope as described previously [24], [25]. The pictures were digitized and analyzed by an image-analysis software (Adobe Photoshop CS3Extended; Adobe, San Jose, CA, USA) to evaluate the cell morphology. As shown in Figure 1, the orientation of each cell was measured as an angle (θ) of the long axis between 0 and 90° with respect to the stretch axis [24], [25]. To estimate the degree of orienting response of the cells, we constructed histograms from more than 100 cell angles where frequency was plotted against orientation.

### Measurement of Cell Area

We measured cell area in order to quantify cell spreading [20], [26], [27], [28]. To calculate the cell area, the cell morphology obtained by phase contrast images was manually outlined. The cell area was displayed in pixel squared. For the evaluation of the cell area, a mean value of the 5 data set was calculated.

### Immunofluorescent Staining

The cells subjected to cyclic stretch or static condition on the silicone chamber were fixed with 4% formaldehyde in phosphate-buffered saline for 1 h at room temperature, and permeabilized with 0.25% Triton X-100 containing 0.5% bovine serum albumin for 1 h [23], [29]. For the detection of microtubules, cells were incubated with a mouse monoclonal anti-α-tubulin antibody (T5168; Sigma, St. Louis, MO, USA) for 8 h and then with an FITC-conjugated anti-mouse secondary antibody (Molecular Probes, Eugene, OR, USA) for 1 h at room temperature for immunodetection. F-actin was stained with rhodamine-phalloidin (Molecular Probes) for 1 h at room temperature. Nuclei were stained with the DNA binding dye, 4,6-diamino-2-phenylindole (DAPI) (Dojin, Kumamoto, Japan). The immunofluorescently stained cells were then visualized by fluorescence microscopy with an imaging-system (BX51 and DP70; Olympus).

### Model Development

Previous studies have established that when ASM cells are subjected to cyclic uniaxial stretching, the actin stress fibers in the direction of stretch dissemble and reassemble in a new direction which is almost perpendicular to the direction of stretching [5], [7]. To understand how the dynamics of microtubules fit into this picture, we developed a model where microtubules and actin stress fibers work in tandem to maintain a homeostatic strain on the actin stress fibers. This hypothesis was based on the following experimental observations. Kaverina et al. [30] found that microtubules actively target and bind to focal adhesions. When a local tensile strain was applied to a focal adhesion, microtubules polymerized in the direction of stretched focal adhesions [31], [32]. Further, local application of actin-myosin contractile inhibitors causes rapid and local depolymerization of microtubules towards the cell center [32]. These results coupled with the observation that cells plated on a 2D substrate maintain a homeostatic strain on the actin stress fibers [33], [34] led us to formulate the notion that microtubule dynamics are linked to the strain on the actin stress fibers. Specifically, under conditions of cyclic stretch, microtubules polymerize and depolymerize in an attempt to maintain a constant homeostatic level of strain across the stress fibers.

#### Cell shape and orientation

We modeled the cell as an ellipse with focal adhesions distributed across its periphery. The orientation of the cell was dependent on the angular density of focal adhesions. Initially, the angular distribution of focal adhesions Π(α) for a cell oriented at an angle θ with respect to the direction of stretch was a Gaussian with mean θ and variance of 5°. When the cell was subjected to cyclic stretch, and focal adhesions dissociate and reform in response to stretch, the orientation of the cell was tracked by using the mode (most probable value) of the angular density function. Similarly, the circularity of the cell was tracked using the circular variance (cv) which is defined as
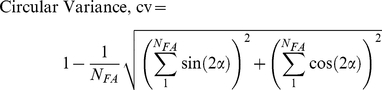
(1)where N_FA_ is the total number of focal adhesions. The cv can take values between 0 and 1 with 1 indicating a circular shape and values closer to 0 indicating a more elliptical shape.

#### Substrate strain

When the cell is subjected to cyclic uniaxial stretch, the strain ε on the substrate in a direction which makes an angle α with respect to the stretch direction varies according to the equation [35], [36]:
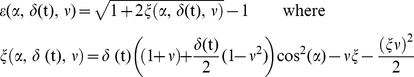
(2)Here δ(t) is the cyclically varying uniaxial strain and *ν* = 0.15 is the Poisson's ratio of the substrate material. It should be noted that for *ν* = 0.15, the absolute value of ε is minimum at an angle of 70° which corresponds to the peak of the cell orientation distribution after 2 h of stretch, suggesting that the cells are aligning in the direction of minimum substrate strain. The value of *ν* was experimentally measured. In our case, the silicone membrane was attached to the sides of the chamber which were much more rigid than the membrane. For a 20% uniaxial strain, the lateral compression was found to be ∼3% and hence the effective value of *ν* was 0.15.

#### Cytoskeletal mechanics

**Figure 2:**
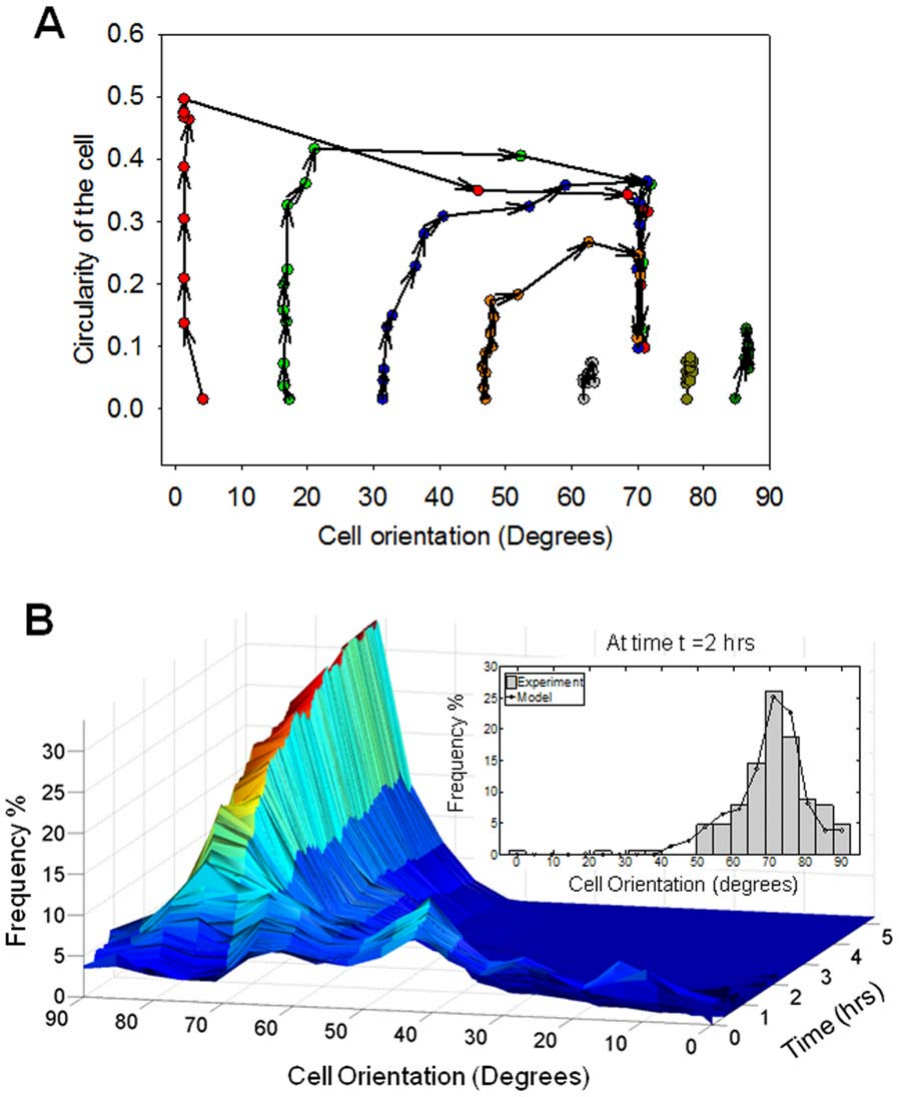
Modeling the Role of Microtubules in Cell Reorientation. (**A**) Model prediction of changes in shape and orientation of cells due to cyclic uniaxial stretching. A cell which was initially oriented parallel with the direction of stretch, first becomes circular before realigning in the direction of minimum strain. The extent to which the cell changes its shape is a function of its initial orientation; with cells that were initially aligned orthogonal to the direction of stretch experiencing very little shape changes due to stretch. (**B**) Histogram of a population of cells with the same initial orientation as the experiment at time t = 0, realigning in response to stretch. It can be seen that the first cells to realign are those that are oriented parallel to the direction of stretch. The inset shows the experimental observed histogram of cell orientations at time t = 2 h with results from the model overlaid on top.

The focal adhesion complex that is connected to the substrate on one end and the cytoskeleton on the other is modeled as a linear elastic spring (K_FA_). Attached to the cytoskeletal end of the focal adhesion complex are two parallel springs K_SF_ and K_MT_ representing the actin stress fibers and the microtubules, respectively. Both actin stress fibers and microtubules are able to support compression and tension and we assign linear elastic properties to K_SF_ and K_MT_. The stiffness of K_SF_ and K_MT_ vary with cyclic stretch as new stress fibers and microtubules attach to and detach from the focal adhesion. The attachment probability of stress fibers was constant independent of strain. However, the attachment probability for microtubules at an angle α is a linear function of the strain on the actin stress fibers at angle α. As the strain on the K_SF_ increases, more microtubules attach to the focal adhesion increasing the stiffness of K_MT_ effectively reducing the strain on the stress fibers. When a microtubule or stress fiber attaches, its initial length is set to 1+ε(α,δ(t),ν), so that the individual fibers that make up K_SF_ and K_MT_ experience different strains at a given time. The detachment probability (P_d_) for individual fibers that make up K_SF_ and K_MT_ is a function of the strain they carry and is given by P_d_ = (1−exp(−*x* ^2^/0.2)), where *x* is the absolute strain on the individual fiber. The above expression approximately corresponds to a breaking threshold which is normally distributed with zero mean and a variance of 10%. It should be noted that the individual fibers that make up K_MT_ and K_SF_ do not break all at once as the initial length of each individual fiber depends on the time the fiber became attached. The stiffness of individual actin microfilaments and microtubules were set based on measured values of 2.6 GPa and 1.7 GPa, respectively [37]. K_FA_ was assumed to be much stiffer than K_SF_ or K_MT_. Also, we assume that the stiffness of the focal adhesions do not change with stretch. The role of K_FA_ is simply to transfer substrate stretch to the cytoskeletal elements. A similar assumption about focal adhesion stiffness was made in a previous study which examined cell reorientation [38]. Although there are experimental data to show that microtubule polymerization is up-regulated when actin stress fibers are strained, the exact functional form is not known. We assumed that the probability of attachment of microtubules increases as a linear function of the strain on the actin stress fibers. Under these assumptions, the only two free parameters that were left to be adjusted to obtain the fit shown in Figure 2B were the mean and variance of the strain threshold at which the microtubules and stress fibers rupture. The values that best fit the data (as shown in Figure 2B) were 0 for the mean and 10% for the variance.

### Statistical Analysis

All data are expressed as means ± standard deviation (SD). Analysis of variance (ANOVA) followed by the Bonferroni test for post hoc analysis or paired *t*-test was used to evaluate the statistical significance (SigmaPlot11.2; Systat Software Inc., San Jose, CA, USA). P<0.05 was considered statistically significant.

## Results

### Cyclic Uniaxial Stretch Induces Cell Reorientation and Alignment of HASM Cells

**Figure 3:**
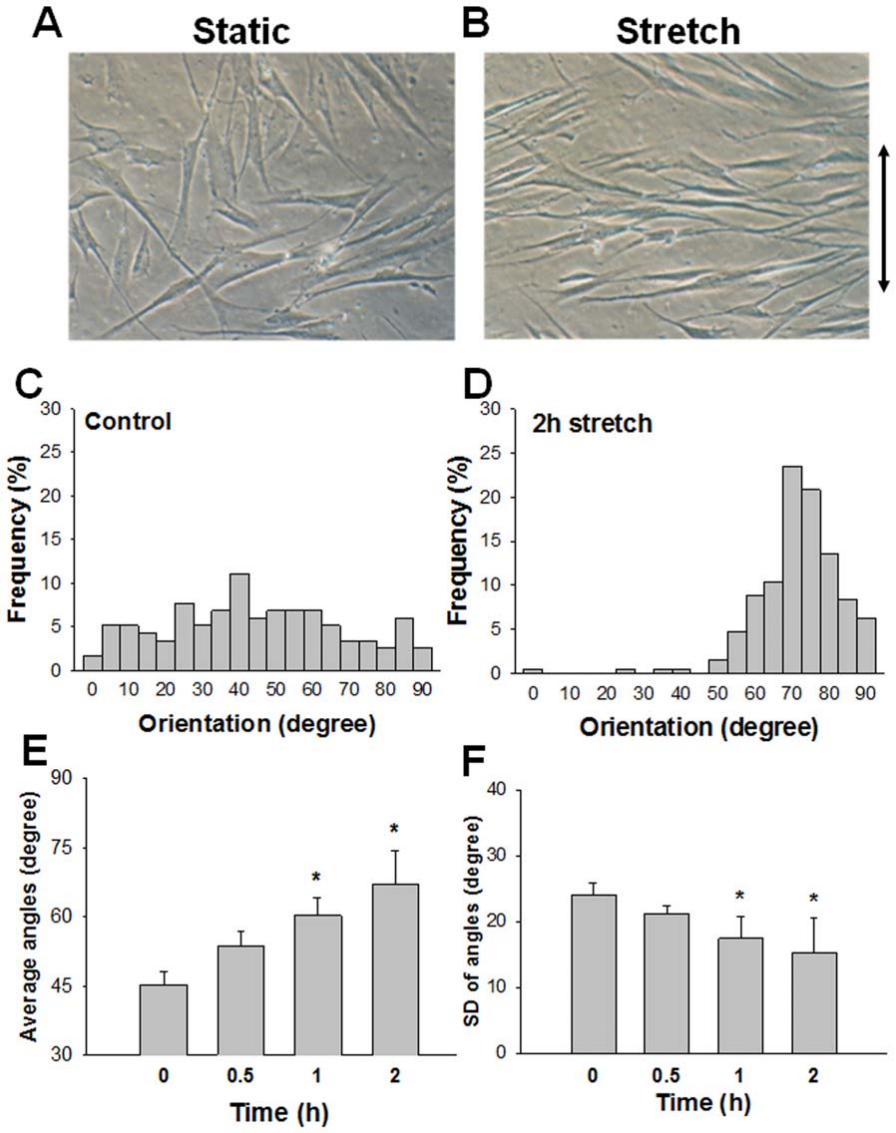
Effects of cyclic stretch on cell reorientation. Phase-contrast images of the cells in the static condition (**A**) or in response to cyclic stretch (20% in strain, 30 cycle/min) (**B**). An arrow indicates stretch direction. Histograms of the orientation response of the cells in the static condition (**C**) or to 2 h uniaxial cyclic stretch (**D**). Images of the unstretched and stretched cells were obtained at 0.5 h, 1 h, and 2 h after the onset of cell stretching. After the cell angles were measured, the angles were binned into 19 groups for every 5°: 0°, 1–5°, 6–10°, …, 81–85°, and 86–90° (**C** and **D**). Total cell number (frequency) was set as 100% and the frequency in each group was expressed relative to the total cell number. (**E** and **F**) The mean and standard deviation (SD) of the cell orientation at each time point. The decreasing SD of angles indicates that the cells were aligning in a direction which was almost perpendicular to the direction of applied stretch. Bar graph represents means ± SD (across 5 different trials). Data were analyzed with one-way repeated-measure ANOVA followed by the Bonferroni test. *: Significantly different (P<0.05) from the unstretched control (time 0) value (n = 5).

We first examined the effect of cyclic uniaxial stretch (0∼2 h) on the orientation of HASM cells. As shown in Figure 1, the orientation of each cell was measured as the angle (θ) of its long axis with respect to the stretch axis [24], [25]. Thus, the angle θ varies between 0 and 90°. In the present study, we report the standard deviation (SD) values to assess the heterogeneity of cell direction. As shown in Figure 3A and 3C, the orientation of unstretched cells was distributed randomly with a mean angle of 43° and SD of 24°. After 2 h of stretch, the cells showed significant reorientation away from the direction of stretch (Figure 3B). As the histogram of cell orientations (Figure 3D) shows, most cells were aligned at an angle close to 70° and the population of cells below 40° was significantly reduced. The mean and SD values of 2 h-stretched cells shown in Figure 3D were 69° and 11°, respectively. The variation in mean and SD of cell orientation from 5 independent histograms is shown in Figure 3E and 3F. The SD bars in Figure 3E reflect variation in the average angle across five independent experiments. Figure 3F reflects the variation in angles within experiments (heterogeneity), averaged over five experiments. The average angles significantly increased in a time-dependent manner (P<0.001, n = 5) (Figure 3E). In contrast, the SD of angles significantly decreased at 1 h (P = 0.005, n = 5) and 2 h (P<0.001, n = 5) (Figure 3F), demonstrating the alignment of ASM cells toward a direction perpendicular to the stretch.

### Stretch Induces F-actin and Microtubule Alignment

**Figure 4:**
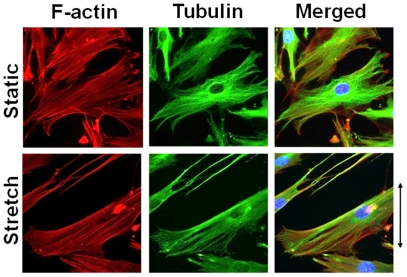
Fluorescent images of organization of F-actin and microtubules in the static or stretched cells. Cells were in the static condition (*upper panels*) or subjected to 2 h uniaxial cyclic stretch (*lower panels*). F-actin was visualized with rhodamine-phalloidin (red). Microtubules were visualized with FITC conjugated secondary antibody following immunostaining with anti-α-tubulin antibody (green). Cell nuclei were stained with DAPI (cyan).

Cyclic stretch induced F-actin stress fiber formation along the axis of cell elongation compared with the unstretched cells (Figure 4). Cyclic stretch also aligned polymerized tubulin along the axis of cell elongation (Figure 4). Polymerized tubulin was accompanied by stress fiber formation both in stretched and unstretched cells.

### Stretch Induced Spreading of Cells

**Figure 5:**
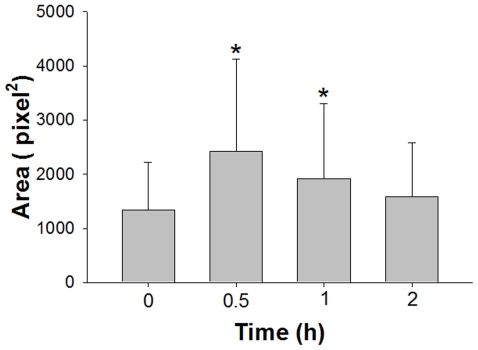
Stretch-induced spreading of cells. Cell spreading was quantified by measuring the area of cells from phase contrast images of cells at time 0, 0.5 h, 1 h, and 2 h after the start of stretch. Bar graphs represent means ± SD (n = 4). *: Significantly different (P<0.05) from the value with unstretched controls (time 0).

Cell spreading was quantified by measuring the area of cells (A_t_) from images of cells fixed at time t = 0, 0.5 h, 1 h, and 2 h after the start of stretch (Figure 5). At 0.5 h, A_0.5_ increased to 1.8 times its value at time 0, A_0_ (P<0.001). At 1 h, A_1_ decreased to 1.43A_0_ (P<0.05) and at 2 h, there was no statistical difference between A_2_ and A_0_ (Figure 5). This indicates that the period before cell alignment is marked by a significant increase in cell spreading.

### Dynamics of Microtubules Are Essential for Stretch-induced Cell Reorientation

**Figure 6:**
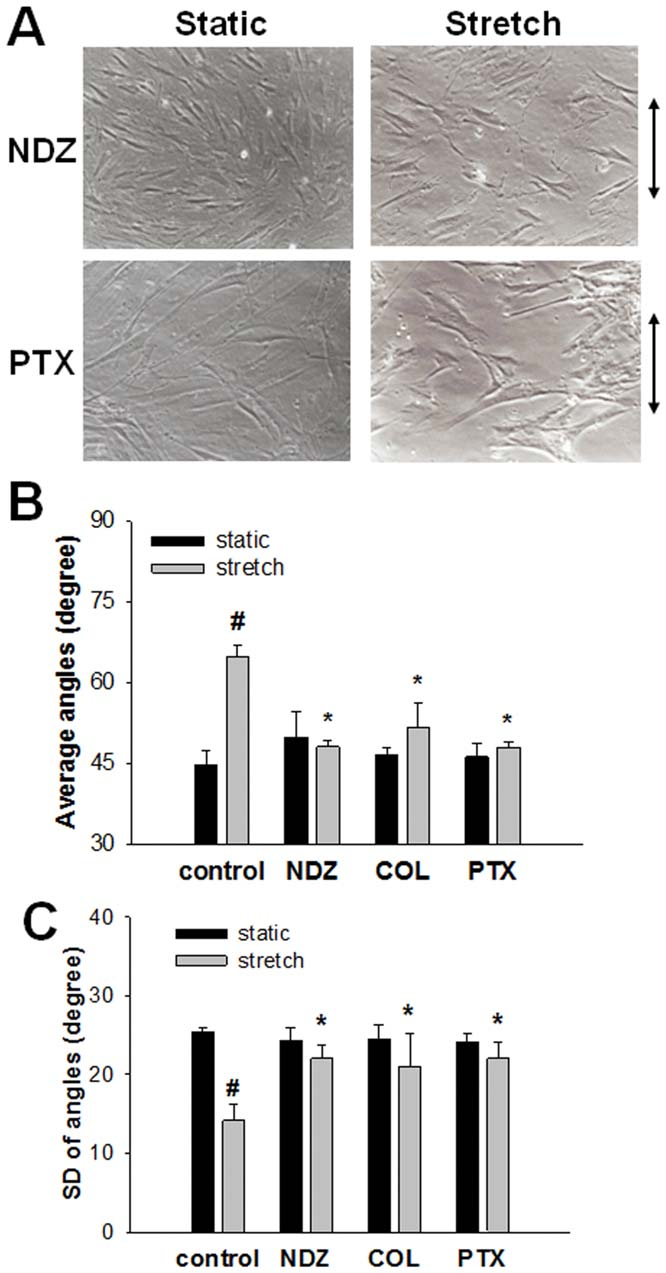
Roles of microtubules in stretch-induced cell reorientation. Cyclic stretch (20%, 2 h) was applied to the cells after pretreatment with either 1 µM nocodazole (NDZ), 10 µM colchicine (COL), or 1 µM paclitaxel (PTX). (**A**) Representative phase-contrast images of the static (*left panels*) and stretched (*right panels*) cells pretreated with either 1 µM nocodazole (NDZ) (*upper panels*) or 1 µM paclitaxel (PTX) (*lower panels*). Average (**B**) and SDs (**C**) of angles of the cell orientation. Bar graphs represent means ± SD (n = 4). *: Significantly different (P<0.05) from the value with cyclic stretch. #: Significantly different (P<0.05) from the value with unstretched control.

Effects of inhibitors of microtubule polymerization, nocodazole and colchicine, or a microtubule stabilizer paclitaxel were examined. Representative phase-contrast images show that the microtubule-related agents, nocodazole (1 µM) and paclitaxel (1 µM), affected the changes in distribution of cell reorientation induced by 2 h cyclic stretch (Figure 6A) compared with the untreated control cells (Figure 6A and 6B). The mean cell orientation in response to 2 h cyclic stretch was significantly lower in the cells treated with nocodazole, colchicine, or paclitaxel than in the stretched cells without inhibitors (P<0.01, n = 4) (Figure 6B). The baseline cell orientation without stretching was not affected by 2 h treatment with any of the drugs (Figure 6B). The SD of angles in response to 2 h cyclic stretch was significantly higher in the cells treated with nocodazole, colchicine, or paclitaxel than in the stretched cells without inhibitors (P<0.01, n = 4) (Figure 6C).

**Figure 7:**
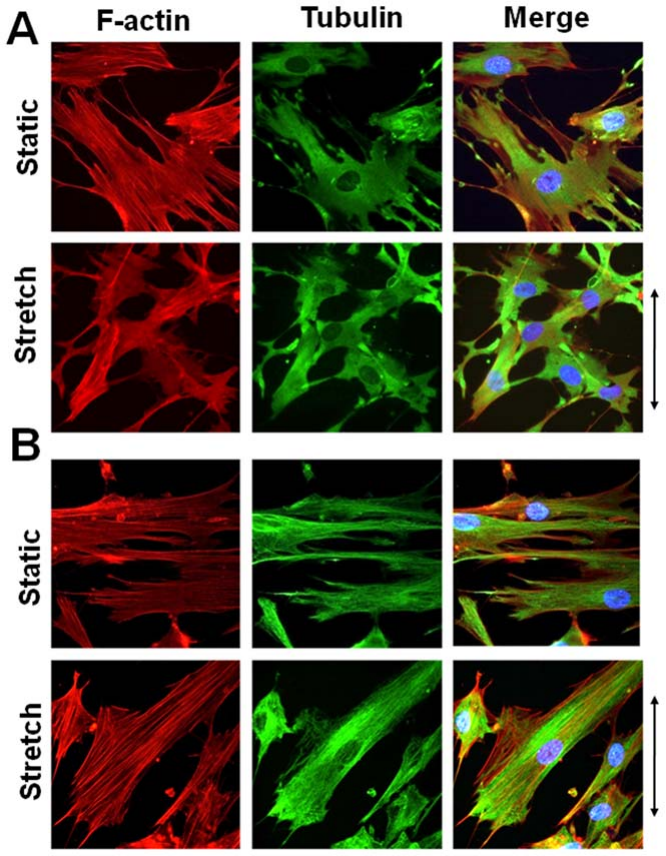
Effects of nocodazole and paclitaxel on the organization of F-actin and microtubules. Fluorescent images of the organization of F-actin stained with rhodamine-phalloidin (red) and microtubules immunostained with anti-α-tubulin antibody (green) in the static or stretched cells. Cell nuclei were stained with DAPI (cyan). The cells were pretreated with either 1 µM nocodazole (NDZ; **A**) or 1 µM paclitaxel (PTX; **B**). Arrows indicate stretch direction.

Next, effects of nocodazole (1 µM) or paclitaxel (1 µM) on organization of F-actin and microtubules in response to 2 h cyclic stretch were investigated. Compared with the control cells shown in Figure 4, formation of tubulin polymerization was blocked by nocodazole treatment both in the unstretched and stretched cells (Figure 7A). In contrast, F-actin formation was not affected by nocodazole in the unstretched cells. When the cells treated with nocodazole were stretched for 2 h, F-actin formation was still observed but did not align (Figure 7A). Formation of tubulin polymerization was preserved by paclitaxel treatment both in the unstretched and stretched cells (Figure 7B). Polymerized tubulin was observed along with F-actin fiber formation both in the unstretched and stretched cells pretreated with paclitaxel (Figure 7B).

### Modeling the Role of Microtubules in Cell Reorientation

To better understand the intracellular dynamics that lead to the reorientation of HASM cells, we developed a model in which microtubules polymerize and reinforce focal adhesions in response to an increase in strain on stress fibers. The simulations suggest that when HASM cells experience cyclic uniaxial stretch, the cells first become circular then elongated in the direction of minimum strain (Figure 2A). The equilibrium angle that the orientation of cells approaches corresponds to the direction of minimum strain which depends on the Poisson's ratio of the underlying substrate (Eq. 2). Further, in a population of randomly oriented cells, the cells that were initially oriented parallel to the strain become more circular than cells which had an initial orientation closer to the direction of equilibrium angle (Figure 2B). The time for an individual cell to reorient also depends on its initial orientation. Cells which were initially parallel to the stretch direction are the first to reorient whereas cells aligned closer to the equilibrium orientation take longer time to reorient (Figure 2B). As the stretch proceeds, the randomly oriented cells tend to align with the degree of alignment increasing with the duration of stretch. After 2 h of stretch, the model is able to match the experimentally observed distribution of cell orientations. However, the model also predicts that stretching cells for longer periods would lead to a more tightly aligned population of cells.

## Discussion

In this study we found that microtubules play a major role in the alignment of HASM cells subjected to cyclic uniaxial stretch. A population of randomly oriented HASM cells showed statistically significant alignment by 1 h but this was preceded by significant cell spreading at 0.5 h. Since it has been established that cell spreading involves microtubules [20], [26], [27], [28], the above finding indicates that microtubule dynamics should contribute to the stretch-induced cell reorientation in HASM cells. Furthermore, when the microtubule assembly was inhibited by colchicine or nocodazole treatment, and also when microtubules were stabilized by paclitaxel treatment, the HASM cells showed no tendency to align since cell orientation remained uniform after 2 h of stretch. To quantitatively understand individual cell dynamics that lead to these experimental observations, we developed a model in which microtubule polymerization and attachment were regulated by mechanical stretch. Together with our experimental findings, these results demonstrate, for the first time, that microtubules play a significant role in the cyclic stretch-induced cytoskeletal remodeling and subsequent cell reorientation in HASM cells.

Contradictory results have recently been reported in NIH3T3 fibroblasts by Goldyn et al [39]. They observed that NIH3T3 cells can align in response to stretch even after knocking out microtubules. Similarly, Hayakawa et al. reported that the cyclic stretch-induced cell alignment was not affected by pharmacological disruption of microtubule polymerization in A10 smooth muscle cell line [40]. These findings suggest that there may be cell type specific differences in the way cells remodel their cytoskeleton in response to cyclic uniaxial stretching. Even though the conclusions by Goldyn et al. [39], [41] indicate that cell re-orientation is purely a consequence of actin remodeling, they report several observations which are similar to ours. In their study, upon stretching non-treated, unstretched cells, both microtubules and F-actin re-align significantly. Further, when treated with cytochalasin D, microtubules no longer align with stretch like they do in untreated cells. More importantly, they found that microtubules can modulate the kinetics of the stretch-induced alignment of NIH3T3 fibroblasts [41]. These observations support our hypothesis that the remodeling of actin in response to stretch is coupled to that of the microtubules.

In this study, we used nocodazole and colchicine to disrupt microtubules. Both drugs are also known to cause an increase in myosin phosphorylation, which may lead to the contractility of actin stress fibers [42], [43], [44]. However, treatment of cells with nocodazole did not induce apparent F-actin formation in HASM cells (Figure 7). Further, we have demonstrated that upon inhibiting microtubule dynamics by treatment with paclitaxel HASM cells fail to align (Figure 6). This implies that it is not just the presence or absence of microtubules, but the dynamic nature of the polymerization process of microtubules, in response to stretch that is critical for the cell to be able to adapt to cyclically varying uniaxial stretch. Further evidence for the involvement of microtubule dynamics may be found in our measurements of projected cell area which is an indicator of how well cells spread [26], [27], [28], [45]. Our results indicate a highly significant increase in cell spreading after 0.5 h of stretch (Figure 5). Spreading progressively decreased until 2 h at which point the projected areas were statistically the same as those of unstretched cells (Figure 5). These results suggest that the reorientation process is preceded by a period of increased microtubule polymerization-depolymerization turnover and cell spreading.

It is known that microtubules can bear compressional stress in cells [42], [46]. On the extracellular side, the focal adhesions bind to extracellular matrix proteins through integrins and on the cytoplasmic side they link to actin stress fibers through various adaptor proteins. It has been shown that microtubules actively target and bind to focal adhesions [30]. Moreover, microtubules and stress fibers are linked by structural and biochemical processes [19]. When a local tensile strain was applied to a focal adhesion, microtubules polymerized toward the focal adhesion that was strained [31], [32]. Putnam et al. [47], [48] demonstrated that 10% static stretch of rat aortic smooth muscle cells significantly increased tubulin polymerization. Nevertheless, the relationship between the dynamics of microtubule polymerization and cyclic stretch has not been fully explained. It has been reported that the speed of tubulin polymerization within a cell is below 10 µm/min [49]. In contrast, the speed of tubulin rapid de-polymerization, called a catastrophe, is much faster than polymerization speed [50]. In the present study, 20% cyclic stretch was applied to the ASM cells at 30 cycle/min. Thus, it is likely that the stretching/unloading speed was sufficiently fast so that polymerization/de-polymerization of tubulin could not complete within one stretching-unloading cycle. Instead, cell shape slowly changed towards cell alignment due to cytoskeletal reorganization and focal adhesion remodeling during repeated cyclic stretching [13], [14], [39]. As shown in Figure 4, cyclic stretch induced microtubule reorientation which was accompanied by actin stress fiber formation. Similar observations of stretch-induced microtubule reorientation have been reported previously in various cell types [39], [40], [51], [52]. Oakley and Brunette [53] demonstrated that microtubules are the first to align prior to cell alignment in cultured fibroblasts. These findings also indicate an important role of microtubule dynamics in regulation of cyclic stretch-induced cell alignment in HASM cells. Furthermore, there is a link between dynamics of microtubules and the fluctuations in mechanical forces on the stress fiber/focal adhesion assembly due to applied cyclic stretch.

Previous studies have demonstrated an important role of experimental conditions, specifically strain, frequency, duration, and cell types, in the cell reorientation induced by cyclic stretch [13], [14], [23], [39], [40], [41], [54]. We previously reported that cyclic stretching primary cultured human pulmonary microvascular endothelial cells (20%, 12 h) induced cell alignment perpendicular to stretch axis [23]. In the present study, we used relatively shorter stretch duration (within 2 h) in order to minimize genomic effects such as protein synthesis. In our preliminary results, stretch-induced alignment behavior of HASM cells was not different between 2 h and 3 h stretch, indicating that 2 h stretch was long enough for primary HASM cells to align. We also found that HASM cells still aligned when the cells were cyclically stretched at a lower (10%) strain for 2 h. Kaunas et al. [14] examined the strain-dependency of stress fiber alignment induced by cyclic stretch (10%, 60 cycles/min, 6 h) in bovine aortic endothelial cells. They found that the stress fiber aligned at 3% strain but not at 1% strain. They developed a model which was capable of describing stress fiber reorganization in response to diverse temporal and spatial patterns of cyclic stretch in bovine aortic endothelial cells [55]. However, the threshold of strain levels which induces cell alignment remains unclear in HASM cells and examining the threshold stain is beyond of the focus of the present study.

Mathematical models have often been employed to help elucidate the mechanism of cell alignment in response to cyclic stretch [34], [55], [56]. Hsu et al. [55] developed a dynamic stochastic model of frequency-dependent stress fiber alignment induced by cyclic stretch in endothelial cells. They concluded that when the rate of stretch is faster than the rate of stress fiber self-adjustment, the stress fibers gradually accumulate and align in a direction that require the smallest deviation form equilibrium. This is the reason why the cell aligns perpendicular to the stretch axis in response to uniaxial stretch at relatively high rate (>0.1 Hz). Thus, if the rate of stretch is faster than the rate of tubulin self-adjustment as well as stress fiber adjustment, cells align perpendicular to the stretch axis in HASM cells.

In constructing the model, we used the experimental observation that microtubules polymerize in response to stretch and depolymerize when tension on actin/myosin is inhibited to form a hypothesis that microtubule dynamics are regulated by the strain on the stress fibers. An alternative hypothesis, based on the above experimental evidence, is that they are responding to stress/strain on focal adhesions. However, we choose the former interpretation as this would imply that the cell is attempting to reestablish the homeostatic pre-stress levels that existed before stretch. This is more likely as the pre-stress in the cell is an important regulator of a variety of cellular functions [57]. The model based on this assumption was able to reproduce the observed dynamics of reorientation (Figure 2). Specifically, our model predicts that HASM cells parallel to the direction of stretch are the first to align, with cells closer to the equilibrium angle taking more time to re-align (Figure 2A). Further, we show that the process of alignment is accompanied by shape changes in the cell. The extent of this shape change also depends on the initial orientation of the cell with cells parallel to the direction of stretch becoming almost circular and then re-elongating in a new equilibrium direction of stretch [13], [58].

Apart from their traditional role in an unstretched cell as compression bearing elements, in our model, microtubules under conditions of cyclic stretch are capable of bearing both compression and tension. This is a plausible assumption as microtubules are polymers that can buckle [59], [60]. Further, live imaging of cyclically stretched cells shows that microtubules straighten out under application of tensile stretch [60]. However, even the simpler Poisson statistics used here are able to reproduce the statistics of cell alignment well.

In summary, our experiments and modeling results demonstrate that microtubules play a major regulatory role in the way HASM cells respond to uniaxial, cyclically varying stretch patterns. Specifically, we find that cell alignment results from the interplay of microtubules and actin stress fibers acting in tandem to maintain a homeostatic pre-strain in the cytoskeleton. These results can help better understand how ASM cells respond to pathological alterations in the mechanical properties of the underlying extracellular matrix induced by the onset of diseases such as asthma.
